# Investigation on the Coaxial-Annulus-Argon-Assisted Water-Jet-Guided Laser Machining of Hard-to-Process Materials

**DOI:** 10.3390/ma16165569

**Published:** 2023-08-10

**Authors:** Yuan Li, Shuiwang Wang, Ye Ding, Bai Cheng, Wanda Xie, Lijun Yang

**Affiliations:** 1 School of Mechatronics Engineering, Harbin Institute of Technology, Harbin 150001, China; yuanli@hit.edu.cn (Y.L.); wsw1999@stu.hit.edu.cn (S.W.); chengbaiidx@163.com (B.C.); 13840372779@163.com (W.X.); 2 Key Laboratory of Micro-Systems and Micro-Structures Manufacturing, Ministry of Education, Harbin Institute of Technology, Harbin 150001, China

**Keywords:** water-jet-guided laser, coaxial-annulus-argon-assisted atmosphere, machining capacity, hard-to-process materials

## Abstract

In this study, the novel coaxial-annulus-argon-assisted (CAAA) atmosphere is proposed to enhance the machining capacity of the water-jet-guided laser (WJGL) when dealing with hard-to-process materials, including ceramic matrix composites (CMCs) and chemical-vapor-deposition (CVD) diamond. A theoretical model was developed to describe the two-phase flow of argon and the water jet. Simulations and experiments were conducted to analyze the influence of argon pressure on the working length of the WJGL beam, drainage circle size, and extreme scribing depth on ceramic matrix composite (CMC) substrates. A comparative experiment involving coaxial annulus and helical atmospheres revealed that the coaxial annulus atmosphere disrupts the water jet proactively, while effectively maintaining the core velocity within the confined working length and enhancing the processing capability of the WJGL beam. Single-point percussion drilling experiments were performed on a CMC substrate to evaluate the impact of machining parameters on hole morphology. The maximum depth-to-width ratio of the groove and depth-to-diameter ratio of the hole reached up to 41.2 and 40.7, respectively. The thorough holes produced by the CAAAWJGL demonstrate superior roundness and minimal thermal damage, such as fiber drawing and delamination. The average tensile strength and fatigue life of the CMCs specimens obtained through CAAAWJGL machining reached 212.6 MPa and 89,463.8 s, exhibiting higher machining efficiency and better mechanical properties compared to femtosecond (194.2 MPa; 72,680.2 s) and picosecond laser (198.6 MPa; 80,451.4 s) machining. Moreover, groove arrays with a depth-to-width ratio of 11.5, good perpendicularity, and minimal defects on a CVD diamond were fabricated to highlight the feasibility of the proposed machining technology.

## 1. Introduction

The aviation and aerospace industries are experiencing rapid development, which has posed significant challenges for the performance of critical components. In response, hard-to-process materials, such as CMCs and diamond, have emerged as viable alternatives to conventional structural materials. However, owing to the distinctive mechanical, thermal, and chemical properties, these materials can hardly be processed by conventional mechanical drilling, grinding, electrical discharge machining, and laser machining with both high efficiency and quality [1,2,3]. Ultrafast laser machining is a proper choice, but its machining capacity is limited when dealing with a structure characterized by a large depth-to-depth/diameter ratio. Hence, it is essential to discover a general and reliable machine tool.

Water-jet-guided laser (WJGL) processing was first introduced by Richerzhagen through the approach of using a compact water jet as a multimode fiber to take advantage of both water jetting and laser machining [4]. Since its invention, this technology has found numerous applications in diverse fields, such as aviation, aerospace, semiconductor, and medical industries. For instance, Sun et al. used a WJGL to cut carbon-fiber-reinforced plastics substrate [5]. Through the multi-pass scanning, a sawtooth shape on the side wall can be observed which results from the perturbance of jet flow induced by the kerf shape. Although the formation of a heat-affected zone is suppressed, the cutting efficiency is relatively low compared with laser machining. Drilling experiments on SiC-reinforced aluminum metal matrix composites and thermal-barrier-coated alloy were put forward by Marimuthu et al. [6,7]. The holes are characterized by a good entrance circularity and low taper. By comparing the numerical and experimental results, it is indicated that when the hole depth increases, the water layer deposited at the hole bottom and substrate surface can induce the local breakup of the WJGL beam, resulting in the discontinuous interaction between the WJGL beam and the substrate, as well as a reduced material removal rate. An in-depth investigation of the surface-structure formation during the WJGL machining of a 718-nickel-based superalloy was carried out by Liao et al. [8]. Their discoveries indicate that the deposited water and confined plasma plume can trigger the appearance of recast layer, which can hardly be removed and further influences the mechanical property of the substrate.

Although WJGL has proved its feasibility in handling some materials, its machining capacity is still troubled by a typical technical bottleneck. Specifically, the water jet stability is easily interrupted by the ambient and deposited water layer, leading to the breakage of the water jet and leakage of the laser energy, which has a serious detrimental impact on the ablation process. Our previous research attempted to solve this problem by using a coaxial helical gas atmosphere [9]. The argon yields a longer working length for the WJGL beam and has a better scribing quality compared to other gases, but the machining efficiency is still limited.

In this paper, a novel coaxial annulus argon atmosphere is first introduced with the purpose of protecting the velocity core of the WJGL beam and promoting its machining capacity. The theoretical model describing the coaxial annulus argon atmosphere and water jet two-phase flow is established. The influence and related mechanism of argon pressure on the working length of the WJGL beam, drainage circle size, and extreme scribing depth on the CMC substrate are revealed, while helical atmosphere is selected as the comparison object. In this study, single-point percussion drilling experiments were carried out on a CMC substrate to uncover the influence of machining parameters on structure morphology and the extreme depth-to-diameter ratio of holes. In addition, through layer-by-layer concentric-circle drilling experiments and tensile tests on CMC specimens, the mechanical property of holes processed by the CAAAWJGL and ultrafast lasers was quantitatively evaluated. On these bases, the CAAAWJGL was applied to scribe micro grooves on CVD diamond, with a large depth-to-width ratio, good consistency, and limited defects.

## 2. Numerical Setup

**Figure 1:**
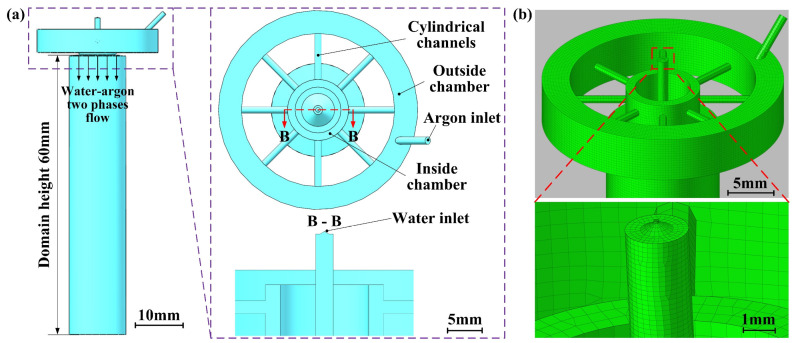
Calculation domain of water–argon two-phase flow: (**a**) three-dimensional structure and (**b**) its mesh generation.

The theoretical basis for characterizing the flow field of the CAAAWJGL is provided in the Supplementary Materials [10,11,12]. A three-dimensional asymmetrical model is established to demonstrate the argon–water-jet two-phase flow, as illustrated in Figure 1a. The argon phase enters the outer chamber through a 1.5 mm diameter inlet and is subsequently directed into the inner chamber through eight cylindrical channels, with a 45° angle between adjacent channels. Meanwhile, the water phase is introduced into the computational domain through a nozzle with an 80 μm diameter. Finally, the argon phase is ejected from the inner chamber, coaxially with the water jet, resulting in a two-phase argon–water-jet flow. Both the water and argon inlets are defined as pressure inlets, and the bottom of the computational domain is defined as the pressure outlet, while all other boundaries are considered to be walls. The external pressure is set at atmospheric pressure. Additionally, unstructured meshes are automatically generated using the tetra/mixed method. To ensure simulation accuracy and computational efficiency, the distance between the bottom of the inside chamber and calculation domain is set as 60 mm. The meshes surrounding the water inlet are refined due to the diameter of the water jet being within three orders of magnitude shorter than the height of the calculation domain, as shown in Figure 1b. It is important to note that the distance between the water inlet and the bottom of the inside chamber is set to 8 mm, which is consistent with the dimension in the coupling device.

The simulation process is reasonably simplified based on the following assumptions:

(1) The dynamic viscosities and densities of water and argon phases are considered to be constants.
(2) The absorption of laser energy during its transmission in the water jet is disregarded, and the internal temperature of the calculation domain is held constant at 300 K.
(3) The non-slip boundary is applied between the fluids and the wall. Additionally, the velocity gradient of fluids is assumed to be perpendicular to the wall.
(4) In the initial stage, the calculation domain is filled with ambient air, meaning that the initial volume fractions of water and argon are both zero.

## 3. Experimental Setup

**Figure 2:**
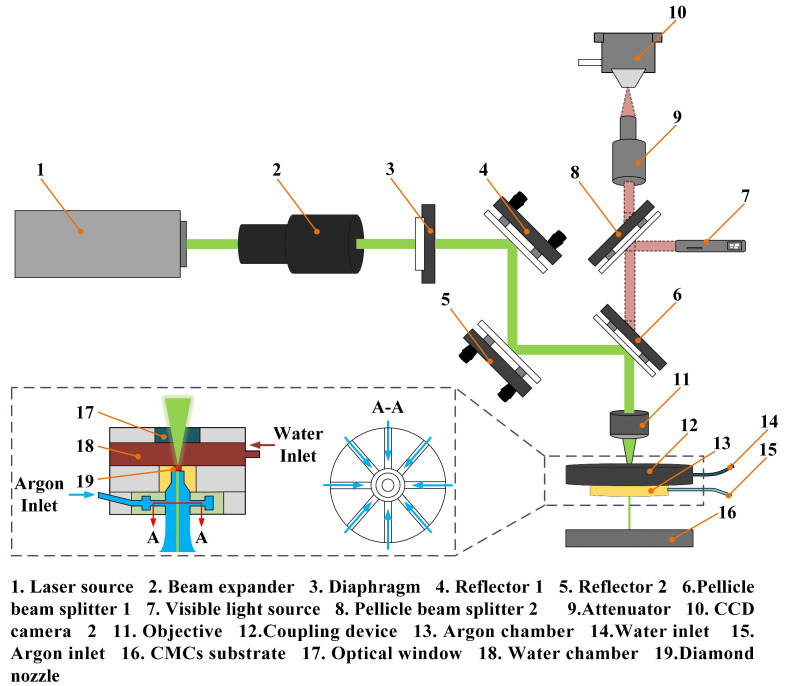
Schematic of the CAAAWJGL machining system.

The laser source utilized for the experiments is a Nd:YAG nanosecond laser (Pulse 532-50-LP, Inngu Laser Co., Ltd., Soochow, China), and the main output parameters are presented in Table 1. The schematic of the experimental setup for CAAAWJGL machining is demonstrated in Figure 2. After beam expansion, the laser beam is focused onto the diamond nozzle through an objective (Linos Focus-Ronar, *f* = 63 mm). The diameter of the laser beam is 15 mm after beam expansion. The theoretical focal spot diameter can be calculated based on Equation (1):

(1) $$D_{C}=\frac{4\cdot \lambda_{L}\cdot f_{O}}{\pi \cdot d_{E}}\cdot M^{2}$$

where *λ*_L_ is the emitted laser wavelength; *f*_O_ is the focal length of the objective; *d*_E_ is laser beam diameter after expansion; M^2^ is the laser-beam quality factor; and *D*_C_ is calculated as 34.13 μm, which is far smaller than the nozzle diameter (80 μm). Therefore, a well-distributed CAAAWJGL can be realized.

**Figure 3:**
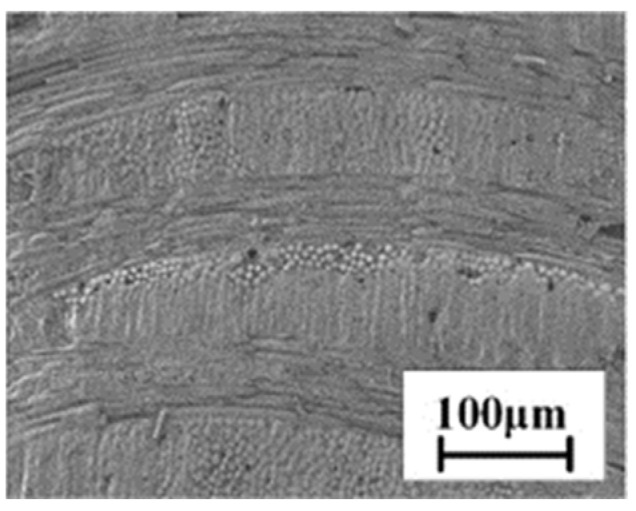
The cross-sectional morphology of the pristine CMC substrate.

The working liquid utilized in this study is deionized water (Watson). The argon gas exhibits high purity levels of 99%, with a dynamic viscosity of 22.624 μPa·s. The CMC substrates and specimens for tensile testing were obtained from the Manufacturing Technology Institute of Aviation Industry Corporation of China (Beijing, China). The CMC substrate’s thickness and porosity are 3.3 mm and 6.2%, respectively. The microscopic cross-sectional morphology of the substrate is depicted in Figure 3.

## 4. Results and Discussions

### 4.1. The Influence of Coaxial Annulus Argon Atmosphere on WJGL Beam

**Figure 4:**
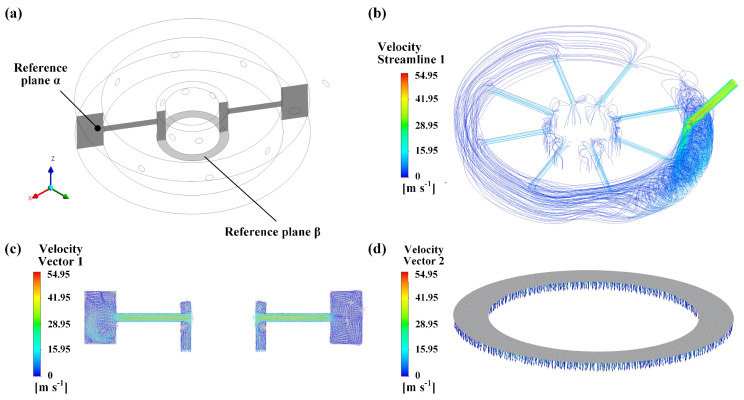
(**a**) Demonstration of reference planes α and β; (**b**) the overall streamline of argon, velocity vector distribution of argon on reference planes (**c**) α and (**d**) β.

**Figure 5:**
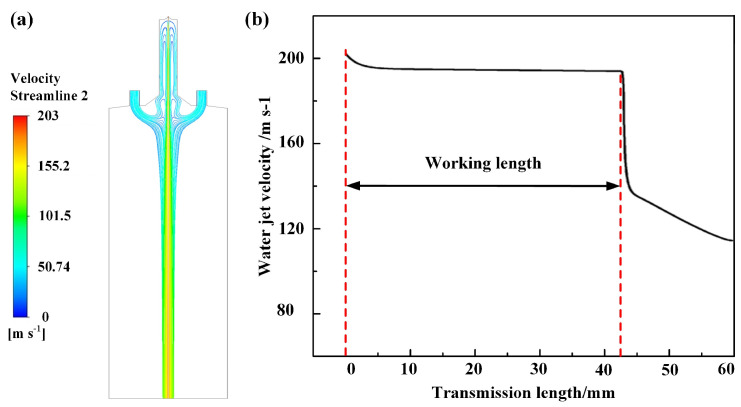
(**a**) Streamline of water–argon two-phase flow in the central top section; (**b**) water jet velocity along axis.

Figure 4 presents the characteristics of the streamline and velocity of the argon phase within the argon chambers at inlet pressures of 20 MPa and 0.1 MPa for the water and argon phases, respectively. The streamline of water–argon two-phase flow on the central top section of reference plane α is shown in Figure 5a. Notably, despite the unilateral and oblique flow of the argon phase into the outside chamber, it exhibits a quasi-uniform flow into the inside chamber through eight cylindrical channels, as displayed in Figure 4b. Upon impinging the inner wall of the inside chamber, the velocity vector of the argon phase changes direction to become vertical, leading to a significant momentum loss, as depicted in Figure 4c. Eventually, the argon phase is ejected from the chamber in an annulus-shaped distribution that encircles the water jet, generating a shield, as shown in Figure 4d. Based on Figure 4 and Figure 5, the maximum value of *M* (relative description is provided in the Supplementary Materials) is calculated as follows:

(2) $$M_{\max}=\frac{1.784\times 50.74^{2}}{997\times 190.3^{2}}=0.00013$$

The velocity of the water jet along its central axis is displayed in Figure 5b, above, exhibiting a gradual decrease to approximately 190.3 m/s. This velocity can be maintained within the transmission length of 42.5 mm, thereby constraining the majority of the water phase within a circular region. The surrounding argon atmosphere is effectively barred from intruding into this region, enabling the laser beam to propagate along the water jet in a state of total internal reflection without any leakage. Based on the aforementioned explanations and the *M*_max_ value, this section constitutes the optimal zone for WJGL machining and is, hence, referred to as the working length. However, when the transmission length exceeds 42.5 mm, the water jet velocity experiences a sudden decline within 5 mm, followed by a gradual reduction. This phenomenon indicates the initiation of water jet divergence, resulting in the gradual disappearance of the jet core beyond the working length. This portion of the jet is unsuitable for WJGL machining due to its severe divergence and unstable velocity.

**Figure 6:**
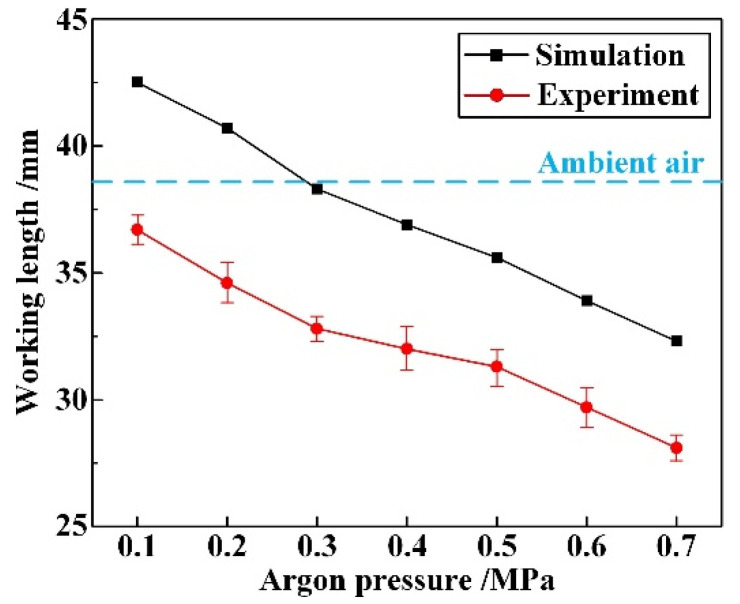
Comparison of working length of water jet obtained via simulations and experiments.

**Figure 7:**
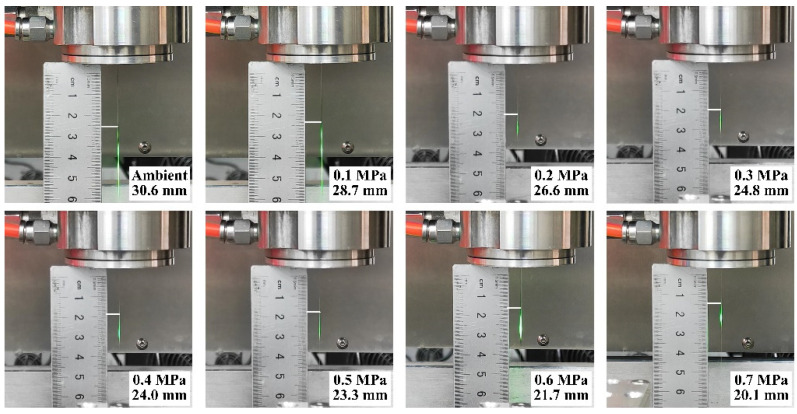
Typical CAAAWJGL beam morphologies and external working-length measurement results.

Figure 6 presents the working lengths of water jet under various argon pressures, as obtained from both numerical simulations and verification experiments. For the latter, a laser power of 2.2 W is used to facilitate the visualization of the working length of the water jet. Digital-camera images (captured by D90, Nikon Corporation, Tokyo, Japan) of typical morphologies are shown in Figure 7. The end of the working length is defined as the point at which the laser beam started to leak from the water jet. It should be noted that the length of the empty region on the ruler is measured as 5 mm. The working length, defined as the sum of the external stable length and the distance between the nozzle exit and bottom of the coupling device (8 mm), was used for all experiments. Each experiment was repeated five times to ensure the accuracy and reliability of the results.

Referring to Figure 6 and Figure 7, above, there exists a non-negligible difference between the numerical and experimental working lengths. This discrepancy can be attributed to the following three factors: Firstly, due to the limitations of the processing technology, the utilized diamond nozzle may exhibit defects such as notches, cracks, or burrs on its entrance and inner surface. These imperfections can trigger disturbances in the water jet as it is ejected from the nozzle. Secondly, the water in the coupling chamber is introduced by the piston pump through the high-pressure pipeline, and this process inevitably brings in impulses, causing certain turbulence. Moreover, impurities and air bubbles may be entrained into the water jet during its transmission and ejection. All of these factors contribute to the reduction in the working length of the water jet obtained from the experiments.

**Figure 8:**
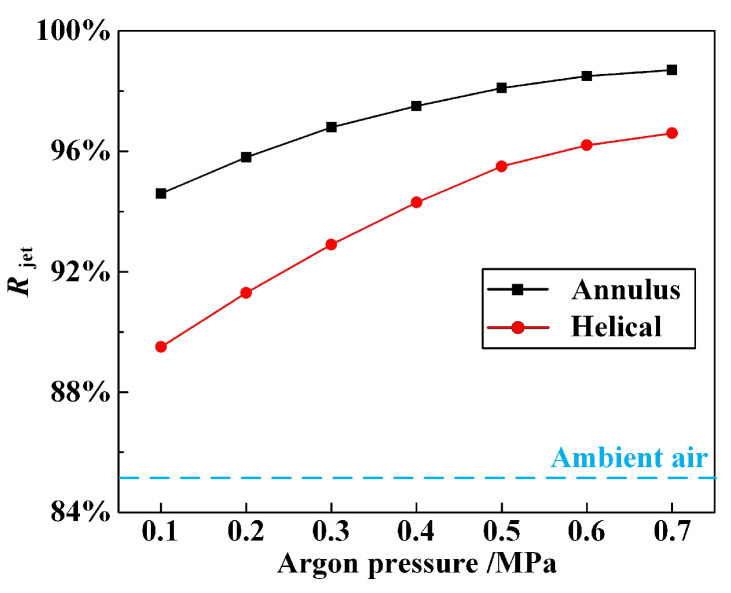
Comparison of *R*_jet_ under different argon atmospheres obtained by numerical simulations.

Furthermore, it is evident that the coaxial annulus argon atmosphere has a detrimental effect on the working length of the WJGL beam. Furthermore, this effect becomes increasingly significant with a higher argon pressure. This finding contrasts with our previous research, which utilized a helical argon atmosphere [9]. Nevertheless, it is noteworthy that the annulus argon atmosphere can ensure that the water jet maintains its core velocity, as compared to the helical atmosphere. To quantitatively characterize this phenomenon, the concentration ratio of the water jet, *R*_jet_, throughout the working length is introduced in this paper, and it is defined as follows:

(3) $$R_{\mathrm{jet}}=\frac{D_{0.85}}{D_{\mathrm{jet}}}\times 100\%$$

where *D*_jet_ is the water jet diameter at the end of working length, and *D*_0.85_ is the circle diameter within which the water jet velocity exceeds 85% of the maximum value. The comparison of *R*_jet_ under coaxial annulus and helical argon atmospheres calculated by the numerical simulation is illustrated in Figure 8, with a fixed external working length and inlet water pressure of 16 mm and 20 MPa, respectively.

**Figure 9:**
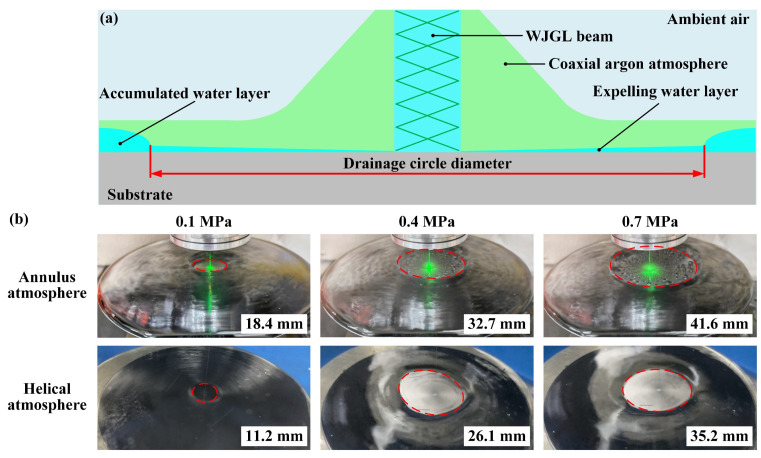
(**a**) Schematic of drainage circle formation. (**b**) Comparison of drainage-circle diameters under different argon atmospheres.

**Figure 10:**
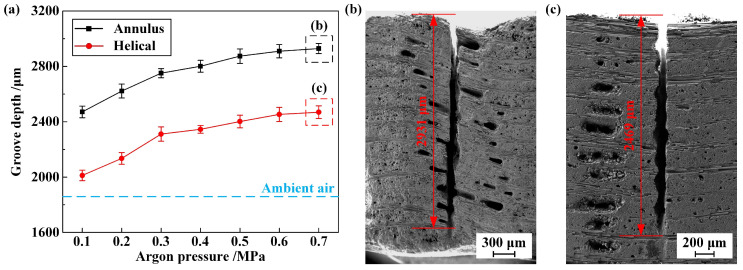
(**a**) Evolution of groove depth under different argon atmospheres. Cross-sectional morphology of groove at extreme depth under (**b**) annulus and (**c**) helical atmospheres.

Verification experiments are proposed to provide insight into the aforementioned discoveries from two perspectives. Firstly, upon the incidence of the WJGL beam being put onto the substrate, an accumulated water layer manifests in the proximity of the water jet. The presence of coaxial argon atmosphere causes the rapid discharge of this water layer around the water jet, leading to the formation of a drainage circle [9], the schematic of which is demonstrated in Figure 9a. The diameters of the drainage circle under different argon atmospheres are characterized and illustrated in Figure 9b. A single-side polished silicon wafer is selected as the substrate to emphasize the morphology of the drainage circle. Additionally, single-row scribing experiments were conducted to reveal the machining capacity of the WJGL beam under different argon atmospheres. The scribing parameters are listed in Table 2. The evolution of the groove depth, as well as the morphologies of the groove at extreme depths, is demonstrated in Figure 10.

Figure 8, above, clearly demonstrates that the assistance of annular argon atmosphere can ensure the maintenance of the core velocity of the water jet when compared with the helical atmosphere. This effect is particularly pronounced at higher argon pressures. Figure 9 and Figure 10, above, provide further evidence of this phenomenon. In Figure 9, coaxial annular and helical argon atmospheres result in a noticeable increase in the drainage-circle diameter, with the former showing a superior performance. In Figure 10, both coaxial annular and helical argon atmospheres significantly enhance the machining capacity of the WJGL beam. To quantitatively analyze the promotion level, the promotion percentage of the groove depth, *P*_gd_, is defined as follows:

(4) $$P_{\mathrm{gd}}=\left(\frac{H_{\mathrm{Ar}}}{H_{\mathrm{air}}}-1\right)\times 100\%$$

where *H*_Ar_ and *H*_air_ are the groove depth obtained under argon atmosphere and ambient air, respectively. The maximum *P*_gd_ under annulus and helical atmospheres is calculated as 57.84% and 32.96%, and the maximum depth-to-width ratio of the groove realized under annulus atmosphere reaches up to 41.2. Additionally, a positive correlation is observed between the promotion level and the argon pressure, but the rate of increase gradually decreased, which is similar to Figure 8.

**Figure 11:**
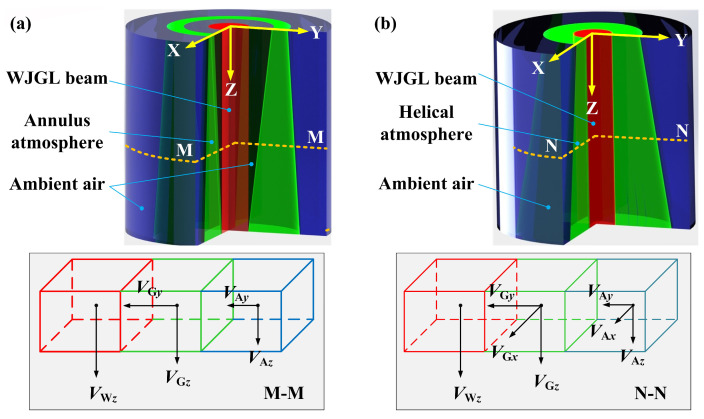
Schematic of velocity vector distribution at the boundary layer units between different fluids near the nozzle exit under coaxial (**a**) annulus and (**b**) helical argon atmospheres.

To comprehensively analyze the numerical and experimental results, boundary layer units at the interface between different fluids near the nozzle exit were selected. The results showed that the argon phase formed a compact shield around the WLGL beam immediately after its ejection. This shield was characterized as a low swirl flow, with a maximum curl of 0.0382 in both atmospheres, based on the simulation results, indicating that the entrainment of the argon phase into the WJGL beam is negligible throughout the working length [13]. Figure 11 illustrates the velocity vectors of each phase over a very short transmission distance in the presence of different forms of argon atmosphere. Here, *V* represents the velocity; subscripts G and A represent the auxiliary argon and external air phases, respectively; and subscripts *x*, *y*, and *z* denote the direction of the velocity vector. In Figure 11a, the air phase adjacent to the WJGL beam and annulus argon shield is expelled, while the external air phase acquires velocity vectors along the Y and Z directions. On the other hand, in Figure 11b, the helical shield continuously interacts with the WJGL beam and the external air phase, leading to the external air phase acquiring velocity vectors along the X, Y, and Z directions. Since the velocities of the WJGL beam and argon near the nozzle exit are nearly identical in both atmospheres, the friction and momentum exchange between argon and the WJGL beam, as well as argon and ambient air, are much more moderate under the annulus argon atmosphere. This, in turn, results in a smaller angular deformation rate of the WJGL beam unit at the boundary layer, leading to a reduced viscous force applied to the WJGL beam unit, in accordance with the Newtonian inner friction law.

Therefore, the WJGL beam can maintain its initial morphology for a certain transmission length under an annular atmosphere, resulting in suppressed boundary breakage and higher jet core velocity when compared to the helical atmosphere. Consequently, there is minimal leakage of laser energy from the WJGL beam, and it impinges on the substrates at a higher velocity, which leads to a larger drainage circle [14]. These phenomena promote the machining capacity of the WJGL beam in two ways: (1) by increasing the laser energy intensity impacting the substrate and (2) by improving the water-expelling ability and reducing water deposition in the groove. The former increases the ablation volume of the CMC substrate per laser pulse, especially when the groove remains shallow. The latter provides an appropriate environment for continuous interaction between the WJGL beam and the substrate, which benefits the extension of the groove depth. This tendency is even more pronounced at higher argon pressures, as the annulus argon shield plays a more significant role in enhancing these effects.

**Figure 12:**
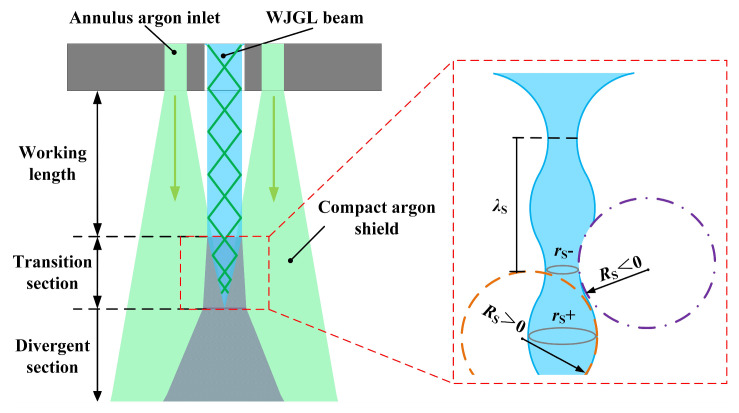
Schematic of WJGL beam interruption by annulus argon atmosphere.

The annulus argon atmosphere not only serves as a shield but also proactively interrupts the transmission of the WJGL beam, leading it to the transition section prematurely, as shown in Figure 12. As referenced in [15], the boundary of the WJGL beam generates a surface wave when transmitting through the gas atmosphere. The interaction between the argon and WJGL beam towards the end of the working length significantly amplifies the amplitude of the surface wave. A disturbance with a wavelength of *λ_s_* applied to the WJGL beam boundary results in radial curvature variation, *r_s_*, and axial curvature variation, *R_s_*. While *r_s_* causes the WJGL beam to diverge more rapidly, *R_s_* restores it to a steady state. The combined effects of *r_s_* and *R_s_* impede the smooth transmission of the WJGL beam, leading to a notable reduction in the working length and leakage of laser energy. This phenomenon is exacerbated under higher argon pressures.

Based on the analyses presented above, the working length of the WJGL beam is shorter under a coaxial annulus atmosphere than under a helical atmosphere. However, the WJGL beam exhibits a stronger machining capacity within this reduced length. To fully capitalize on the benefits of an annulus atmosphere, it is imperative to maintain the working length within a reasonable range.

### 4.2. Single-Point CAAAWJGL Percussion Drilling Experiments

**Figure 13:**
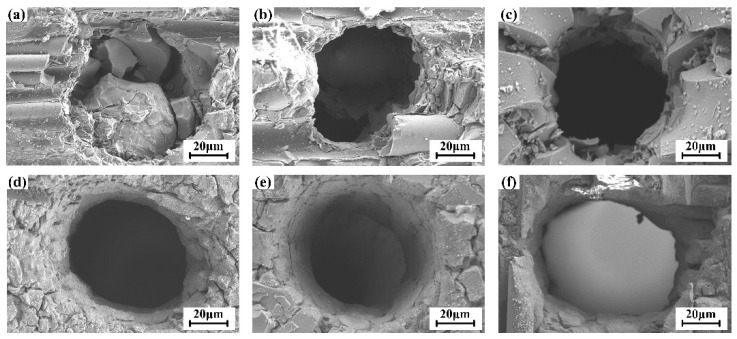
Entrance morphology of holes processed by percussion drilling under water pressure of 20 MPa and pulse energy of (**a**) 0.1 mJ, (**b**) 0.2 mJ, (**c**) 0.4 mJ, (**d**) 0.6 mJ, (**e**) 0.8 mJ, and (**f**) 1.0 mJ.

**Figure 14:**
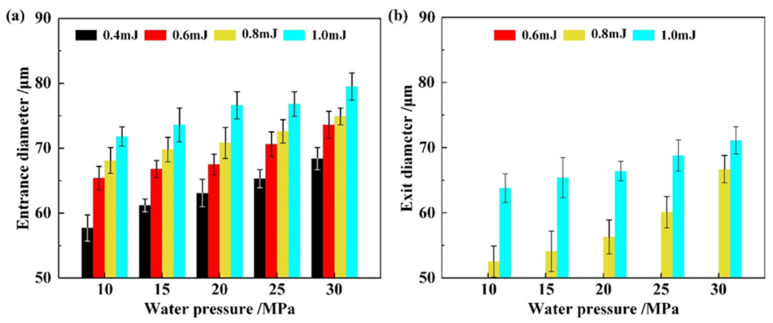
(**a**) Entrance and (**b**) exit diameters of holes under different water pressures and pulse energies.

According to the results in Section 4.1, a coaxial annulus argon atmosphere working at 0.7 MPa was employed to assist the WJGL beam machining of a CMC substrate. The external working length was set as 12 mm. Single-point percussion drilling experiments were conducted to investigate the influence of machining parameters on the hole morphology. The parameters are presented in Table 3. Each experiment was repeated five times. The representative morphologies of the hole entrance are depicted in Figure 13. The statistical results of the entrance and exit diameters of the holes are outlined in Figure 14.

**Figure 15:**
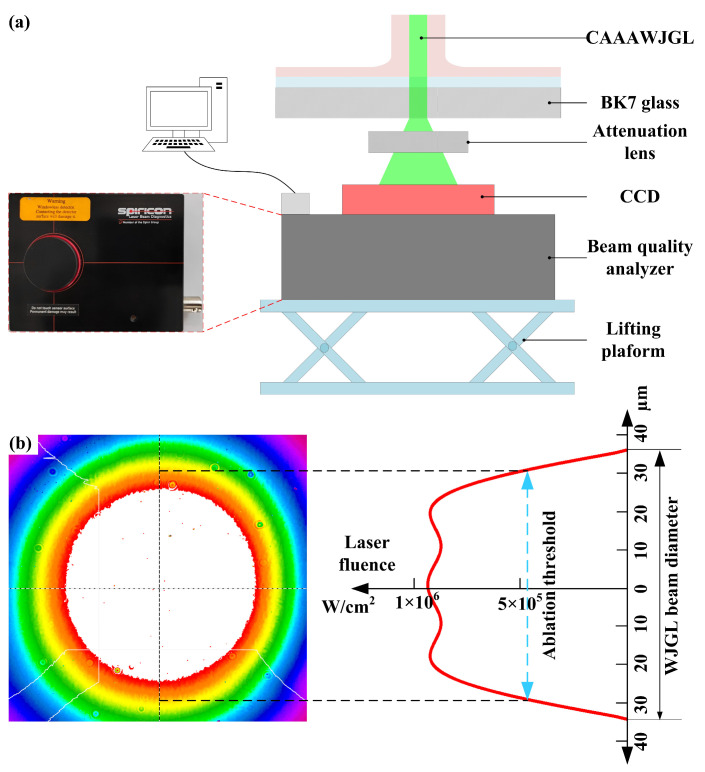
(**a**) Schematic of experimental setup for measuring the laser energy distribution within WJGL beam. (**b**) Measured result and schematic of ablation threshold corresponding to laser fluence.

Insufficient ablation occurs at the surface layer when pulse energies of 0.1 mJ and 0.2 mJ are employed, resulting in an irregular boundary of the hole entrance and unsuccessful drilling; thus, the corresponding hole diameters are excluded in Figure 14, above. To explain this phenomenon, an experimental setup was built to measure the laser energy distribution within the WJGL beam, as illustrated in Figure 15a. The WJGL beam was incident on the BK7 glass, and the laser transmitted through the BK7 glass and attenuation lens and then reached the CCD connecting to the beam quality analyzer (SP620U, Ophir-Spiricon, North Logan, UT, USA). According to Figure 15b, the utilization of low laser energy levels leads to a reduced ablation area that surpasses the substrate’s ablation threshold. Consequently, the overall volume of ablation remains low, even with increasing numbers of laser pulses and water pressures. When the laser pulse energy exceeds 0.6 mJ, a thorough hole forms under all water pressures, yielding a maximum depth-to-diameter ratio of 40.7, an outcome that is difficult to achieve using other machining technologies. This finding underscores the crucial role of laser pulse energy in WJGL machining compared to other parameters. Furthermore, the maximum value of the hole entrance’s diameter is smaller than that of the WJGL beam’s diameter, as supported by Figure 15b. This result implies that the laser energy is constrained within the water jet during the drilling process. Consequently, the ablation is localized, reducing any extra damage on the hole’s side wall.

Moreover, it is worth noting that the SiC fibers at different layers exhibit anisotropic distribution, and the diameters of warp and weft fibers in different positions also deviate from each other, as evidenced by Figure 3. When the laser beam interacts with these fibers inhomogeneously during the CAAAWJGL drilling process, the porous structure within the substrate may cause the laser beam to break up locally, resulting in the beam being reflected to an unpredictable location instead of transmitting downwards. These phenomena pose significant obstacles to achieving uniform ablation of the substrate and the formation of circular holes.

### 4.3. Layer-by-Layer Inside-Out CAAAWJGL Concentric-Circle Drilling and Tensile Tests

**Figure 16:**
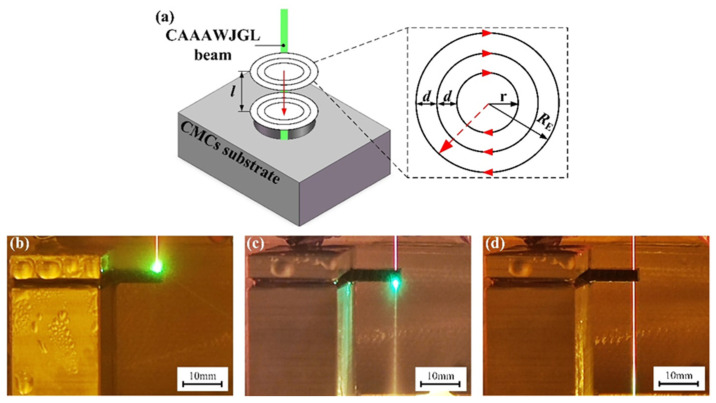
(**a**) Schematic of layer-by-layer inside-out concentric-circle drilling path. Digital morphology of drilling process, (**b**) drilling initialization, (**c**) hole penetration, and (**d**) end of drilling process.

**Figure 17:**
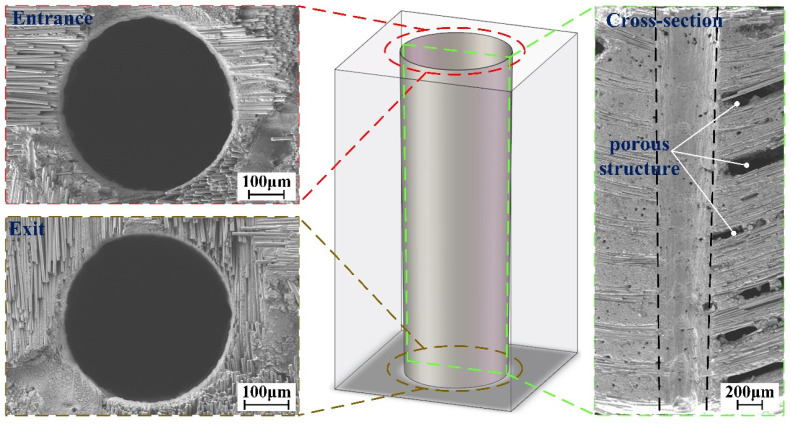
Typical vertical-hole morphology of CMC sample.

**Figure 18:**
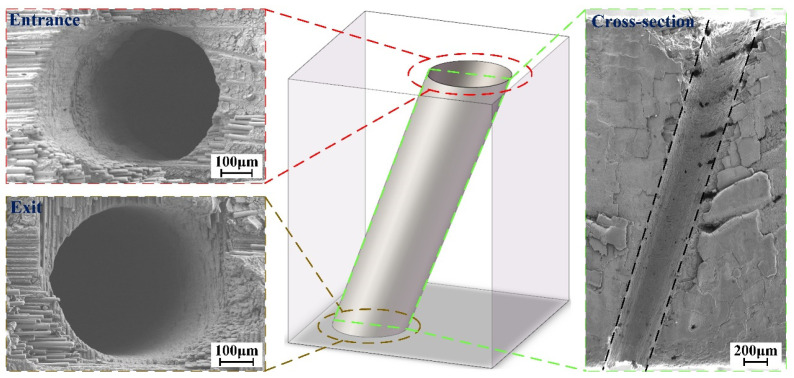
Typical inclined-hole morphology of CMC sample.

The application of CMCs in aeroengine combustors requires microhole drilling that satisfies three critical requirements: (1) a diameter in the range of hundreds of microns, (2) a good mechanical property, and (3) limited machining defects. To address these requirements, a layer-by-layer inside-out concentric-circle drilling strategy was proposed in this study [16], as shown in Figure 16a. The drilling progress at different stages is presented in Figure 16b–d, where a laser safety glass is used to enhance the clarity of the drilling process. Vertical and inclined holes were fabricated on CMC specimens, using the parameters listed in Table 4. Each experiment was repeated five times. The characteristic dimensions of the holes are listed in Table 5 and Table 6. The typical entrance, exit, and cross-sectional morphologies of the holes are depicted in Figure 17 and Figure 18.

It is evident that both holes exhibit excellent circularity at the entrance and exit. Machining defects such as fiber drawing, microcracks, delamination, and heat-affected zones are nearly nonexistent in the cross-sectional view. Furthermore, the taper of the inclined hole is smaller than that of the vertical hole. The morphological deviation can be attributed to differences in the drainage channel. Specifically, when drilling the inclined hole, the substrate is positioned at an angle. The combined effect of gravity and the argon atmosphere facilitates the smooth expulsion of the water layer on the substrate surface and within the hole, creating an optimal environment for the effective interaction between the WJGL beam and the substrate, consequently resulting in a larger exit diameter. In contrast, when drilling the vertical hole, the drainage of the water layer relies solely on the argon atmosphere.

**Figure 19:**
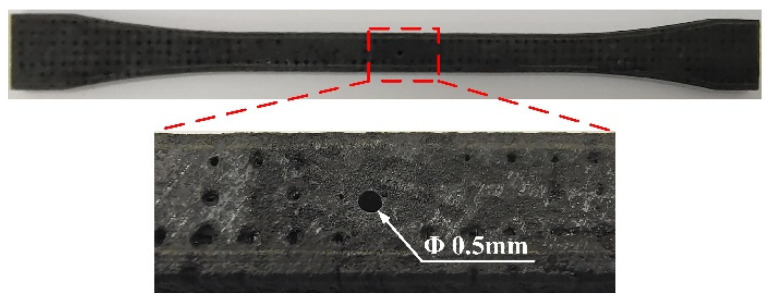
Morphology of CMC specimen for tensile test processed by CAAAWJGL.

**Figure 20:**
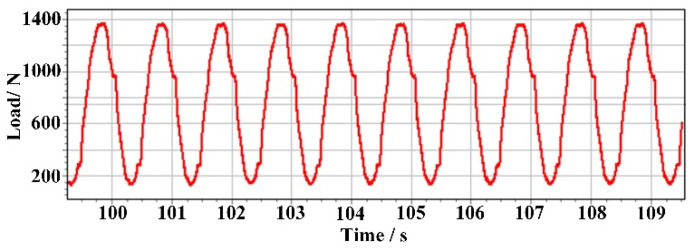
Periodic load applied to the CMC specimen in the tensile test.

Tensile tests were carried out to quantitatively evaluate the mechanical property of the microhole processed by the CAAAWJGL. A thorough hole with a diameter of 0.5 mm was fabricated on the center of the specimen according to GJB 6475-2008 (a China a Chinese military standard) [17], using the parameters listed in Table 3, as illustrated in Figure 19. To highlight the advantage of CAAAWJGL machining, picosecond-laser (GX-30, Edgewave, San Diego, CA, USA) and femtosecond-laser (Pharos-15, Light Conversion, Vilnius, Lithuania) machining were chosen as the contrast objects, and the parameters are listed in Table 7. The same drilling strategy was employed. After hole drilling, the specimens were cleaned ultrasonically for 5 min and left to stand for 24 h under room temperature (300 K) and a humidity level of 65%. Then, periodic load was applied on the specimens by an electronic tensile testing machine (AGXplus, Shimadzu, Kyoto, Japan), as shown in Figure 20.

**Figure 21:**
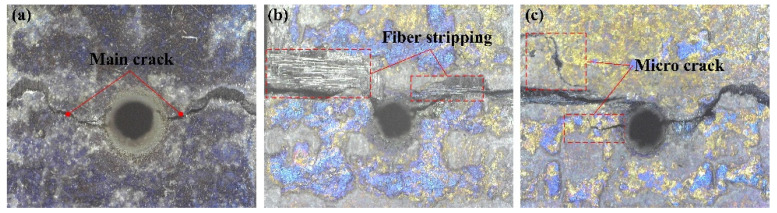
Characteristic specimen morphologies after tensile tests processed by (**a**) CAAAWJGL, (**b**) femtosecond laser, and (**c**) picosecond laser.

The experimental results on the tensile strength and fatigue life of specimens processed by different machining approaches are documented in Table 8 and Table 9, respectively. The characteristic morphologies of the specimens after the tensile tests are depicted in Figure 21. The results indicate that the specimens processed by CAAAWJGL exhibit a superior performance in terms of the tensile strength and fatigue life compared to those processed by ultrafast lasers. The CAAAWJGL-machined specimens demonstrate minimal instances of fiber stripping and microcracks.

Based on the experimental outcomes and discoveries in the existing literature [18], the CAAAWJGL machining technique demonstrates its superiority through its stronger machining capacity and better mechanical property compared to dry ultrafast laser machining. The reasons for this can be elucidated as follows.

The CAAAWJGL machining process involves the simultaneous actions of scouring and cooling via the modified water jet, whereby the debris and confined plasma plume are swiftly expelled by the rapid water jets subsequent to its generation, avoiding the formation of molten deposits on the machined surface. Consequently, the boundary between warp and weft SiC fiber is distinct without any drawing, thereby preserving the mechanical properties of the machined surface. Additionally, the CAA atmosphere effectively removes the accumulated water layer at both the structure bottom and surface, creating a clean environment for the subsequent machining process. Consequently, the WJGL beam can continuously ablate the substrate, ensuring a constant machining capacity throughout the machining process.

In contrast, the dry ultrafast laser machining relies on auxiliary gas to expel the debris, thus demonstrating considerably lower efficacy compared to the high-speed water jet, and it is prone to inducing residual deposition, fiber drawing, and an oxide layer. These machining defects significantly impact the mechanical properties of the machined surface [19]. Moreover, the shielding effect triggered by laser-induced plasma further sets up obstacles for the interaction between laser and substrate, which can hardly be suppressed by the auxiliary gas and leads to the sharp shrinkage of the machining capacity.

### 4.4. Further Application of CAAAWJGL on Scribing CVD Diamond

**Figure 22:**
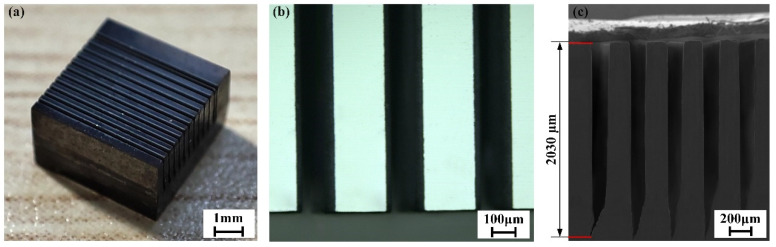
(**a**) The overall, (**b**) top, and (**c**) cross-sectional morphology of grooves on CVD diamond.

In order to extend the application of CAAAWJGL machining to the aviation and aerospace industries, scribing experiments were conducted on 3.5 mm thick CVD diamond (Shanghai Academy of Spaceflight Technology, Shanghai, China). This material is currently employed in satellite heat sinks and is acknowledged as one of the most challenging materials to machine. The scribing strategy is based on our previous research [20], and the scribing parameters are listed in Table 10. The groove morphologies are depicted in Figure 22 and Table 11, demonstrating good perpendicular sidewalls without any signs of turning. Notably, the grooves exhibit a depth-to-width ratio of 11.5, with an average width and depth of 175.8 μm and 2030 μm, respectively. Moreover, typical defects, such as microcracks, edge breakage, carbonization, and large taper, are absent. These results suggest that none of the existing machining technologies is capable of achieving such high-quality and high-efficiency machining of CVD diamond.

## 5. Conclusions

In this study, a coaxial annulus argon atmosphere was proposed to assist the WJGL machining of hard-to-process materials with high quality and efficiency, including CMCs and CVD diamond. The discoveries can be summarized as follows:

(1) The introduction of coaxial annulus argon atmospheres proactively interrupts the water jet, leading to a decrease in the working length. Nevertheless, compared to the utilization of a coaxial helical atmosphere, the CAAAWJGL beam exhibits superior maintenance of its core velocity within the constrained working length, therefore enhancing the processing capability. This enhancement is manifested in a single row scribing depth-to-width ratio of 41.2, representing a noteworthy improvement of 57.84% and 32.96% compared to ambient conditions and a helical atmosphere, respectively.
(2) The modification of the CAAAWJGL beam improves the ablation capacity of single-point percussion drilling on CMCs, obtaining a maximum depth-to-diameter ratio of 40.7. Furthermore, by employing an inside-out strategy, the successful fabrication of vertical and inclined holes is accomplished with exceptional circularity at both the entrance and exit, minimal taper, and negligible fiber drawing and delamination. The CAAAWJGL exhibits superior processing efficiency compared to femtosecond and picosecond lasers, meanwhile achieving a better tensile strength and fatigue life. Furthermore, the feasibility of CAAAWJGL machining is demonstrated through the scribing of CVD diamond with good perpendicularity and minimal defects on CVD diamond.

However, it should be noted that the reduced working length limits the machining flexibility when dealing with components featuring complex curved surface features. Future research will focus on exploring approaches to overcome this technical bottleneck, such as optimizing the gas chamber’s structure and atmosphere composition.

## Figures and Tables

**Table 1:** Main output parameters of applied laser source.

Parameter	Wavelength	Pulse Duration	Repetition Rate	Average Power	Pulse Energy	M^2^
Value	532 nm	100–120 ns	50–100 kHz	50 W (max)	1 mJ (max)	12

**Table 2:** Experimental parameters of WJGL single-row scribing.

Pulse Energy	Repetition Rate	Scribing Velocity	Scribing Time
1 mJ	50 kHz	10 mm/s	200

**Table 3:** Experimental parameters of single-point percussion drilling.

Water Pressure/MPa	Pulse Energy/mJ	Repetition Rate/kHz	Drilling Time/s
10, 15, 20, 25, 30	0.1, 0.2, 0.4, 0.6, 0.8, 1.0	50	60

**Table 4:** Experimental parameters of layer-by-layer inside-out concentric-circle drilling.

Parameter	R_E_/μm	d/μm	r/μm	*l*/μm	Layer Number
Value	210	30	30	505	6
Argon pressure /MPa	Repetition rate /kHz	External working length /mm	Pulse energy /mJ	Path scanning velocity/mm·s^−1^	Drilling time/s
0.7	50	12	1.0	10	67

**Table 5:** Characteristic dimensions of vertical holes.

Hole Number	1	2	3	4	5	Average
Entrance diameter/μm	493.5	487.8	491.1	490.7	488.9	490.4
Entrance circularity/μm	3.5	4.2	4.1	3.6	4.1	3.9
Exit diameter/μm	385.6	383.5	386.9	386.5	385.5	385.6
Exit circularity/μm	5.3	5.2	4.9	4.6	4.5	4.9
Taper	0.0163	0.0158	0.0158	0.0158	0.0157	0.0159
Depth-to-diameter ratio	6.69	6.77	6.72	6.73	6.75	6.73

**Table 6:** Characteristic dimensions of inclined holes.

Hole Number	6	7	8	9	10	Average
Entrance diameter/μm	498.5	499.3	498.1	497.9	498.7	498.5
Entrance circularity/μm	4.2	5.1	4.8	4.7	3.2	4.4
Exit diameter/μm	416.5	424.6	417.5	416.8	425.1	420.1
Exit circularity/μm	5.6	4.8	5.2	5.5	4.4	5.1
Taper	0.0124	0.0113	0.0122	0.0123	0.0112	0.0119
Depth-to-diameter ratio	6.62	6.61	6.63	6.63	6.62	6.62

**Table 7:** Experimental parameters of femtosecond and picosecond laser drilling.

Parameter	R_E_/ Μm	d/ μm	r/ μm	*l*/ μm	Layer Number	Wavelength/nm
Laser						
Femtosecond	230	30	20	165	20	1030
Picosecond	230	30	20	165	20	343
Parameter	Pulse duration/fs	Pulse energy /μJ	Repetition rate /kHz	Path scanning time	Path scanning velocity/mm·s^−1^	Drilling time/s
Laser						
Femtosecond	255	400	37.5	400	150	87
Picosecond	8500	75	300	1100	400	95

**Table 8:** Tensile strength of CMC specimens processed by different machining approaches.

Machining Approach	Specimen 1/MPa	Specimen 2/MPa	Specimen 3/MPa	Specimen 4/MPa	Specimen 5/MPa	Average /MPa
CAAAWJGL	198	237	209	214	205	212.6
Femtosecond	212	182	177	202	198	194.2
Picosecond	183	217	206	185	202	198.6

**Table 9:** Fatigue life of CMC specimens processed by different machining approaches.

Machining Approach	Specimen 1	Specimen 2	Specimen 3	Specimen 4	Specimen 5	Average
CAAAWJGL/s	90,854	87,621	91,215	89,541	88,448	89,463.8
Femtosecond/s	74,510	71,362	70,651	73,021	73,857	72,680.2
Picosecond/s	79,954	81,825	77,514	78,659	80,305	80,451.4

**Table 10:** Experimental parameters of grooves scribing on CVD diamond.

Water Pressure/MPa	Argon Pressure /MPa	Repetition Rate /kHz	External Working Length /mm	Scribing Interval on the Same Layer/μm	Scribing Layer along Depth	Pulse Energy /mJ	Path Scanning Velocity/mm·s^−1^
30	0.7	50	16	20	50	1.0	20

**Table 11:** Characteristic dimensions of grooves on CVD diamond.

Groove Number	1	2	3	4	5	Average
Width/μm	177.8	173.4	173.7	176.8	177.3	175.8
Depth/μm	2030.5	2042.6	2035.8	2032.2	2008.9	2030.0

## Data Availability

The data presented in this study are available on request from the corresponding author.

## Supplementary Materials

The following supporting information can be downloaded at https://www.mdpi.com/article/10.3390/ma16165569/s1, Theoretical basis for characterizing the flow field of CAAAWJGL [10,11,12,21,22].
