# Supplementary material for: Investigation on the Coaxial-Annulus-Argon-Assisted Water-Jet-Guided Laser Machining of Hard-to-Process Materials
Source: Materials (Basel). 2023 Aug 10;16(16):5569. doi: 10.3390/ma16165569 (PMC10456533; doi:10.3390/ma16165569)
Supplement: Supplementary file 1 [file materials-16-05569-s001.zip › materials-2555254-supplementary.pdf]

Supplementary materials

1. Theoretical basis for characterizing the flow field of CAAAWJGL

Coaxial annulus argon atmosphere formed through radial channels are proposed in this work to assist WJGL machining. To validate the feasibility of this method as well as investigate its influence on the stability and consistency of WJGL beam, a theoretical model is built to characterize the argon-water jet two phases flow based on volume of fluid (VOF) model.

According to the law of conservation of mass, the net flow rate of fluid element equals to the variable quantity of its internal mass. Hence, the continuity equation of fluid element can be written as Eq.(1) based on differential analysis method:

$$\frac{\partial \rho_{\mathrm{w,a}}}{\partial t}+\frac{\partial\left(\rho_{\mathrm{w,a}}V_x\right)}{\partial x}+\frac{\partial\left(\rho_{\mathrm{w,a}}V_y\right)}{\partial y}+\frac{\partial\left(\rho_{\mathrm{w,a}}V_z\right)}{\partial z}=0 \tag{1}$$

Where $\rho_{\mathrm{w,a}}$ is the fluid density(subscripts w and a indicate water and argon), V_x,V_y and V_z are the fluid velocity along X, Y and Z axes.

Taking ∇ as fluid divergence, Eq. (1) can be rewritten as:

$$\frac{\partial \rho_{\mathrm{w,a}}}{\partial t}+\nabla\bullet\left(\rho_{\mathrm{w,a}}\mathbf{V}\right)=0 \tag{2}$$

$$\nabla=\frac{\partial}{\partial x}+\frac{\partial}{\partial y}+\frac{\partial}{\partial z} \tag{3}$$

Where $\mathbf{V}$ is the fluid velocity vector.

Assuming that the water, argon and ambient air are incompressible fluids, Eq. (2) can be simplified as:

$$\nabla\bullet\mathbf{V}=0 \tag{4}$$

The momentum theory states that the variation rate of momentum of fluid element equals to the external force applied on it, and the momentum equation of fluid element in the differential form can be described as:

$$\rho_{\mathrm{w,a}}g-\nabla p_{\mathrm{w,a}}+\nabla\tau_{ij}+\mathbf{F}=\rho_{\mathrm{w,a}}\frac{d\mathbf{V}}{dt} \tag{5}$$

Where $p_{\mathrm{w,a}}$, τ_{ij} and $\mathbf{F}$ are the pressure, viscous force and surface tension applied on the fluid element, respectively. Considering that all the fluids involved in this work are incompressible, Eq.(5) can be rewritten as:

$$\begin{cases}\rho_{\mathrm{w,a}}g_x-\dfrac{\partial p_{\mathrm{w,a}}}{\partial x}+\mu\left(\dfrac{\partial^2V_x}{\partial x^2}+\dfrac{\partial^2V_x}{\partial y^2}+\dfrac{\partial^2V_x}{\partial z^2}\right)+F_x=\rho_{\mathrm{w,a}}\dfrac{dV_x}{dt}\\ \rho_{\mathrm{w,a}}g_y-\dfrac{\partial p_{\mathrm{w,a}}}{\partial y}+\mu\left(\dfrac{\partial^2V_y}{\partial x^2}+\dfrac{\partial^2V_y}{\partial y^2}+\dfrac{\partial^2V_y}{\partial z^2}\right)+F_y=\rho_{\mathrm{w,a}}\dfrac{dV_y}{dt}\\ \rho_{\mathrm{w,a}}g_z-\dfrac{\partial p_{\mathrm{w,a}}}{\partial z}+\mu\left(\dfrac{\partial^2V_z}{\partial x^2}+\dfrac{\partial^2V_z}{\partial y^2}+\dfrac{\partial^2V_z}{\partial z^2}\right)+F_z=\rho_{\mathrm{w,a}}\dfrac{dV_z}{dt}\end{cases} \tag{6}$$

Where F_x, F_y, and F_z are the external forces applied on the fluid element, respectively. μ is the

dynamic viscosity of fluid.

To characterize the two phases flow field more precisely, RNG *k*-*ε* turbulence model is employed for the following considerations:

(1) The RNG *k*-*ε* model introduces the time-averaged strain rate R_{sr} into the *ε* equation, which is capable of improving the computational accuracy.

(2) The RNG *k*-*ε* model offers analytical formulas to solve turbulent Prandtl number and fluid viscosity of low Reynolds number flow[1].

The specific transport equation, the prediction equations of turbulent kinetic energy *k*, dissipation rate *ε* and time-averaged strain rate $R_{s,r}$ are expressed as:

$$v_{\mathrm{w,a}} = C_0 \frac{k^2}{\varepsilon} \tag{7}$$

$$\frac{\partial k}{\partial t} + U_j \frac{\partial k}{\partial x_j} = -\overline{u_i u_j} \frac{\partial U_i}{\partial x_j} + \frac{\partial}{\partial x_j}\left(\frac{K_{w,a} \partial k}{\sigma_1 \partial x_j}\right) - \varepsilon \tag{8}$$

$$\frac{\partial \varepsilon}{\partial t} + U_j \frac{\partial x}{\partial x_j} = -C_1 \frac{\varepsilon}{k} \overline{u_i u_j} \frac{\partial U_i}{\partial x_j} + \frac{\partial}{\partial x_j}\left(\frac{K_{w,a} \partial \varepsilon}{\sigma_2 \partial x_j}\right) - C_2 \frac{\varepsilon^2}{k} - R_{sr} \tag{9}$$

$$R_{s,r} = \frac{C_0 \eta^3 (1 - \eta/\eta_0) \varepsilon^2}{(1 + \alpha \eta^3) k} \tag{10}$$

$$\eta = \frac{k}{\varepsilon} \left[\left(\frac{\partial U_i}{\partial x_j} + \frac{\partial U_j}{\partial x_i} \right) \frac{\partial U_i}{\partial x_j} \right]^{1/2} \tag{11}$$

Where $v_{\mathrm{w,a}}$ is the turbulent kinematic viscosity. U_i and U_j are the mean velocity components of fluid element. $K_{w,a}$ is the turbulent viscosity of momentum. $-\overline{u_i u_j}$ is the Reynolds stress tensor. $(\sigma_1, \sigma_2, C_0, C_1, C_2, \eta_0, \alpha)$ are constants and their values are (0.7179, 0.7179, 0.0845, 1.42, 1.68, 4.377, 0.012)[2]. The dynamic properties of two phases flow can be characterized by solving the above equations.

The argon ejects coaxially with WJGL beam, forming annulus distributed argon shield around the beam. The Reynolds number is the key factor that determining the spatial and temporal stability of water-argon two phases flow[3]. The Reynolds number of two phases flow can be described as:

$$\mathrm{Re}_{\mathrm{mix}} = \left(\frac{v_{\mathrm{a}} d_{\mathrm{a}}}{u_{\mathrm{a}}} \right) \left[\left(1 - \frac{d_{\mathrm{w}}^2}{d_{\mathrm{a}}^2} \right) + \frac{d_{\mathrm{w}}^2}{M d_{\mathrm{a}}^2} \right] \tag{12}$$

Where v_{w} and v_{a}, d_{w} and d_{a} are the velocities and equivalent diameters of water and argon phases. u_{a} is the dynamic viscosity of argon. *M* is the momentum flux ratio of fluid element, and it can be expressed as:

$$M = \frac{\rho_{\mathrm{a}} v_{\mathrm{a}}^2}{\rho_{\mathrm{w}} v_{\mathrm{w}}^2} \tag{13}$$

Referring to the research of Villermaux[4], the water jet dominates the two phases flow in the stable length when M is far smaller than 1. However, the assisted gas atmosphere can determine whether the two phases flow will break up when M is much larger than 1. Furthermore, a critical momentum flux ratio M_c exist for two phases flow and its value is 35 according to Rehab[5]. When $M>M_c$, the water jet may diverge immediately at the nozzle. When $M<M_c$, the two phases flow can maintain the stable state within a considerable length. The above analyses explain that the assisted argon pressure should be in a proper range to keep its auxiliary function on WJGL machining.

Reference

[1] Nektarios, K., John, G. B., Nicolas, C. M. (2012). Evaluation of Reynolds stress, k-ε and RNG k-ε turbulence models in street canyon flows using various experimental datasets. *Environmental Fluid Mechanics*, 12, 379-403. https://doi.org/10.1007/s10652-012-9240-9

[2] Yakhot, V., Orszag, S. A., Thangam, S., Gatski, T. B., Speziale, C. G. (1992). Development of turbulence models for shear flows by a double expansion technique. *Physics of Fluids A: Fluid Dynamics*, 4, 1510-1520. https://doi.org/10.1063/1.858424

[3] Lasheras, J. C., Hopfinger. E. J. (2000). Liquid jet instability and atomization in a coaxial gas stream. *Annual Review of Fluid Mechanics*, 32 (1), 275-308. https://doi.org/10.1146/annurev.fluid.32.1.275

[4] Villermaux, E., Rehab, H., Hopfinger, E. J. (1994). Breakup regimes and self-sustained pulsations in coaxial jets. *Meccanica*, 29 (4), 393-401. https://doi.org/10.1007/BF00987574

[5] Rehab, H., Villermaux, E., Hopfinger, E. J. (1997). Flow regimes of large-velocity-ratio coaxial jets. *Journal of Fluid Mechanics*, 345: 357-381. https://doi.org/10.1017/S002211209700637X
